# Lactate Thresholds and the Simulation of Human Energy Metabolism: Contributions by the Cologne Sports Medicine Group in the 1970s and 1980s

**DOI:** 10.3389/fphys.2022.899670

**Published:** 2022-07-22

**Authors:** Henning Wackerhage, Sebastian Gehlert, Henry Schulz, Sebastian Weber, Susanne Ring-Dimitriou, Oliver Heine

**Affiliations:** ^1^ Department of Sport and Exercise Sciences, Technical University of Munich, Munich, Germany; ^2^ Department of Sports Biosciences, Institute for Sports Science, University of Hildesheim, Hildesheim, Germany; ^3^ Chair of Sports Medicine/Sports Biology, Technical University of Chemnitz, Chemnitz, Germany; ^4^ INSCYD GmbH, Salenstein, Switzerland; ^5^ Department of Sport and Exercise Science, Paris Lodron University of Salzburg, Hallein, Austria; ^6^ Olympic Training Centre Rhineland, Cologne, Germany

**Keywords:** simulating energy metabolism, lactate treshold testing, slow component, pyruvate deficit, maximal rate of glycolysis, V̇O_2max_, ν_La max_, Cologne group

## Abstract

Today, researchers, practitioners, and physicians measure the concentration of lactate during a graded exercise test to determine thresholds related to the maximal lactate steady state (maxLass) as a sensitive measure of endurance capacity. In the 1970s and 1980s, a group of Cologne-based researchers around Wildor Hollmann, Alois Mader, and Hermann Heck developed the methodology for systematic lactate testing and introduced a 4 mmol^.^L^−1^ lactate threshold. Later, they also developed the concept of the maxLass, and Mader designed a sophisticated mathematical model of human energy metabolism during exercise. Mader`s model simulates metabolic responses to exercise based on individual variables such as maximum oxygen uptake ( $\dot{V}$ O_2max_) and the maximal rate of lactate formation (ν_La.max_). Mader’s model predicts that the ν_La.max_ reduces the power at the anaerobic threshold and endurance performance but that a high ν_La.max_ is required for events with high power outputs in elite athletes. Mader’s model also assumed before the millennium that the rate of fat oxidation is explained by the difference between glycolytic pyruvate synthesis and the actual rate of pyruvate oxidation which is consistent with current opinion. Mader’s model also simulated the $\dot{V}$ O_2max_ slow component in the mid-1980s. Unfortunately, several landmark studies by the Cologne group were only published in German, and as a result, contributions by the Cologne group are under-appreciated in the English-speaking world. This narrative review aims to introduce key contributions of the Cologne group to human metabolism research especially for readers who do not speak German.

## Introduction

In the 1970s and 1980s, a group of Cologne-based physicians and exercise physiologists made major contributions to lactate testing and the modeling of human exercise metabolism (Wackerhage, 2021). Arguably, their work is under-appreciated outside German-speaking countries. This is largely due to the fact that the Cologne group published many of their studies in German. Moreover, several important publications by the Cologne group are not listed on Medline and so are hard to find. As several of these publications contain major conceptual advances in relation to lactate testing and human exercise metabolism, we decided to summarize the work of the Cologne group in this review.

Before discussing the contributions of the Cologne group, we will first clarify the terminology in relation to threshold concepts. This is necessary, as for example, Heck et al. already listed no less than nine terms for thresholds in 1985 and the number has grown since (Faude et al., 2009). Generally, thresholds are defined either based on standard gas exchange and ventilatory measurements as pioneered by Karlman Wasserman and colleagues (Poole et al., 2020; Keir et al., 2022) or on lactate measurements which was introduced by the Cologne group (Mader et al., 1976; Faude et al., 2009).

There are two types of ventilatory and lactate-based thresholds for which the following terms have been used:

1) Thresholds terms describing the first, exercise-related rise of the concentration of lactate typically during a graded exercise tests. Terms used to describe this include “lactate threshold 1” (LT1) (Jamnick et al., 2020), “anaerobic threshold” (Wasserman et al., 1973), and “aerobic threshold” (Kindermann et al., 1979).
2) Thresholds terms linked to the maximal lactate steady state (maxLass or MLSS, (Heck et al., 1985). In addition to “maximal lactate steady state”, other terms used include “lactate threshold” 2 (LT2) (Jamnick et al., 2020), “aerobic-anaerobic transition or threshold” (Mader et al., 1976), “anaerobic threshold” (Kindermann et al., 1979), or “individual anaerobic threshold” (Stegmann et al., 1981). The maxLass is also related to the concept of “critical power” (Iannetta et al., 2022) which we do not discuss further here.

In this review, we only refer to the second threshold, i.e., the maximal lactate steady state. For this, we use the acronym “maxLass” throughout. However, the maxLass is difficult to measure in practice and before Heck et al. (1985) introduced the concept of the maxLass, Mader et al. (1976) proposed a “4 mmol^.^L^−1^ lactate threshold” essentially as an estimate for the maxLass. In our review, we therefore also use the term “4 mmol^.^L^−1^ lactate threshold” to specifically refer to the 4 mmol^.^L^−1^ lactate estimate of the maxLass. Occasionally, we also use other threshold terms used in specific studies that we cite.

## What Did We Know About Lactate Metabolism and Thresholds Before the First Major Paper by the Cologne Group in 1976?

In 1807/1808, Jöns Jacob Berzelius detected lactate in hunted stags (Gladden, 2008), suggesting that lactate had increased during exercise. In the period that followed, researchers including Nobel laureates August Krogh (Krogh and Lindhard, 1920) and Archibald Vivian Hill (Hill and Lupton, 1923) characterized the response of lactate during exercise. However, this work was descriptive and did not result in standardized, lactate-based, submaximal tests of endurance capacity. Instead, Hill and Lupton conceptualized the $\dot{V}$ O_2max_ as a measure of the cardiorespiratory capacity of an individual (Hill and Lupton, 1923).

The two major limitations of the $\dot{V}$ O_2max_ are that it requires cardio pulmonary exercise testing to exhaustion (CPET) and that it is not a sensitive measure of actual endurance performance. When searching for submaximal measures of endurance capacity, Cologne-based Wildor Hollmann introduced in 1958 the concept of the “point of optimal efficiency of ventilation” which is the lowest ventilation for taking up one liter of oxygen (Hollmann, 2001). Unfortunately, Hollmann’s “point of optimal efficiency of ventilation” from 1958 “did not receive recognition perhaps because much of the research was published in German”, to directly cite the authors of a recent review on exercise intensity thresholds (Poole et al., 2020). The point of optimal efficiency of ventilation is determined by a tangent at the VE curve starting from the origin (Hollmann, 2001). In 1964, Wasserman and colleagues first used the term “threshold” in the form of “threshold of anaerobic metabolism” (Wasserman and McIlroy, 1964) which later became the “anaerobic threshold” (Wasserman et al., 1973). Furthermore, 3 years after Wasserman’s publication of the anaerobic threshold the Cologne group then developed modern lactate testing which we will discuss now.

## Lactate in 20 µl Blood Samples and the 4 mmol^.^L^−1^ Lactate Threshold: The 1976 Paper

The first major study by the Cologne group on lactate testing was published in 1976 in *Sportarzt und Sportmedizin* (Mader et al., 1976), which was the house journal of the West German *Sportärzte* (sports physicians). What was the background of the 1976 study? The first author was Alois Mader, a physician who in 1974 had fled from East to West Germany. In the West, he soon became a member of Wildor Hollmann’s sports medicine group in Cologne. With him he brought a method to measure the concentration of lactate in 20 μl of blood which he had developed together with the chemist Peter Haase in Eastern Germany in 1970. At that time, advances in spectrophotometric and fluorometric methods (Lowry, 1972) made it possible to quickly measure the concentration of lactate enzymatically in microliters of capillary blood from the earlobe. This greatly improved the measurement of lactate as it was previously measured chemically over several hours in milliliters of arterial blood obtained by more risky arterial puncturing (Hollmann, 2001). The 1976 study also gives detailed advice on how to conduct lactate testing by stating that the ergometry should be sports-specific (i.e., cycle ergometry for cyclists, treadmill running for runners, and canoe ergometry for canoeists), the stages during a graded lactate exercise test should last at least 4 min, and the workload or velocity at the 4 mmol^.^L^−1^ lactate threshold reacts more sensitively to changes in sports-specific endurance performance than the $\dot{V}$ O_2max_. Mader et al. (1976) also reported data for the velocity at the 4 mmol^.^L^−1^ lactate threshold, $\dot{V}$ O_2max_, heart volume for students, 5000 m and 10,000 m marathon runners, national team football players, and professional cyclists. This data further supported the idea that the power or velocity at the 4 mmol^.^L^−1^ lactate threshold is a more sensitive biomarker for endurance performance than the $\dot{V}$ O_2max_ or heart volume. Finally, Mader et al. (1976) proposed that training intensity can be classified based on the results of a lactate exercise test. Specifically, they labeled training below 4 mmol^.^L^−1^ of lactate as extensive endurance training, training at the 4 mmol^.^L^−1^ lactate threshold as threshold training, and training above 4 mmol^.^L^−1^ of lactate as intensive endurance training. To our knowledge, this is the first 3-zone training intensity classification. Importantly, whilst Mader et al. proposed a 3-zone training intensity classification, they did not suggest that threshold training was a particularly effective form of endurance training.

**FIGURE 1:**
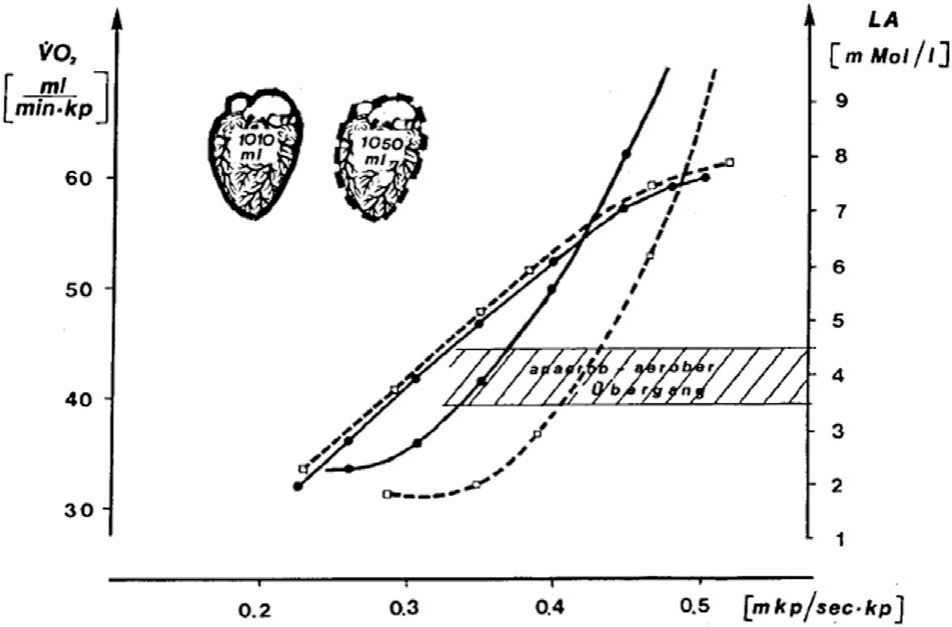
Effect of a ≈6-week endurance training by one individual at the intensity of the 4 mmol^.^L^−1^ lactate threshold (Mader et al. termed it “aerobic-anaerobic transition”) on heart volume measured by X-ray, $\dot{V}$ O_2max_, and the workload at the at 4 mmol^.^L^−1^ lactate threshold. The study also highlights that the exercise protocol (e.g., the duration of each step) affects the lactate curve and that lactate testing should be specific for each sport (e.g., testing rowers on a treadmill has little predictive power). Printed with kind permission from Mader et al. (1976).

In Figure 1, we reproduce a figure of the 1976 study which is largely unknown in the English-speaking world. It shows the effect of a ≈6-week endurance training on heart volume measured by X-ray, the $\dot{V}$ O_2max_, and the workload–lactate relationship. The figure clearly shows that the workload at 4 mmol^.^L^−1^ of lactate is a more sensitive measure of the adaption to endurance training than the $\dot{V}$ O_2peak/max_ or heart volume which changed little from pre to post training.

## After the 1976 Paper: The Concept of the maxLass and the Mathematical Modeling of Energy Metabolism

In the years after 1976, researchers in German-speaking countries and elsewhere routinely performed lactate exercise tests and learned how to interpret the resultant data. At that time, several groups proposed strategies to determine an “individual” lactate threshold instead of a fixed 4 mmol^.^L^−1^ lactate threshold. To find out whether the 4 mmol^.^L^−1^ lactate threshold is at the transition from purely aerobic to partially anaerobic metabolism, Heck and colleagues measured lactate during 30 min exercise bouts at different constant workloads. This work led to the term “maximum lactate steady state”, abbreviated as maxLass or as MLSS by others. Their criterion for the maxLass was the “[maximal] *treadmill speed* [where] *the lactate level does not increase* […] *more than 1 mmol* ^*.*^ *L* ^*−1*^ *during the last 20 min* [of exercise]” (Heck et al., 1985). In contrast to the 1976 study, the 1985 study and the concept of the maxLass (or MLSS) are discussed today in the English-speaking literature.

At the beginning of the 1980s, Mader then started to develop a model of human energy metabolism during exercise. He first introduced his model in his habilitation, a type of thesis that used to be a prerequisite for a professorship, in 1984. It described a mathematical model that simulates the exercise response of ATP, phosphocreatine, lactate, pH, glycolytic ATP resynthesis, and ATP resynthesis by oxidative phosphorylation in humans (Mader, 1984). Mader not only described the underlying formulas that connect the pathways of human energy metabolism but also used them to create a computer model.

What inspired Alois Mader to develop such a mathematical model of human exercise metabolism, what influenced its design and how did it work? Mader realized that human energy metabolism in the 1980s was a black box. Specifically, researchers investigated the effect of exercise on the concentration of blood lactate but the mechanisms by which an exercise input causes a metabolic output were unclear. The challenge was to come up with a set of equations that linked input and output.

Key ideas for his model also originate from the Austrian scientist Ludwig von Bertalanffy, one of the founders of systems biology (von Bertalanffy, 1950). Bertalanffy had developed a theory for open systems such as living organisms. Such open systems exchange food and gases with the environment and maintain a steady state. This is different from closed systems that do not exchange molecules with the environment (von Bertalanffy, 1950). Mader then applied the principles of open systems and his knowledge of metabolic reactions to quantitatively model the behavior of human energy metabolism in response to exercise. When designing his model, he also followed *Occam*’*s razor* principle which requires choosing the simplest theory to try to explain a phenomenon. This implies using as few variables and equations as possible and to link them to each other through clear, logical, mathematical relationships (Walsh, 1979).

## Mader’s Mathematical Model of Human Exercise Metabolism

Here, we describe Mader’s mathematical model of human exercise metabolism. Mader first published this model in German (Mader, 1984), then together with Heck in English (Mader and Heck, 1986), and a revised English version in 2003 (Mader, 2003). While the latter studies were published in English, they are hard to follow as, for example, the 2003 study contains no less than 33 mathematical equations. To make Mader’s reasoning and model accessible, we will now discuss the main features of Mader’s model without mathematical formulas.

The starting point for Mader’s mathematical model was to convert exercise intensity or power output in Watts into the rate of ATP hydrolysis in mmol^.^L^−1^ of ATP per second, assuming a constant work efficiency. Because there is only ≈5–8 mmol ATP per kg muscle (Harris et al., 1974; Dawson, 1983), any exercise-induced rise in the rate of ATP hydrolysis must immediately be matched by increases in the overall rate of ATP resynthesis to avoid ATP depletion and *rigor mortis*.

ATP resynthesis occurs *via* three reactions which are

1) ATP resynthesis from phosphocreatine (PCr) *via* the Lohmann reaction (ADP + phosphocreatine ↔ ATP + creatine),
2) ATP resynthesis *via* glycolysis which leads to pyruvate and lactate synthesis and a reduction of the pH,
3) ATP resynthesis from oxidative phosphorylation which is linked to oxygen uptake.

Moreover, Mader has calculated the rate of lactate oxidation as the group of George Brooks had earlier reported that lactate oxidation or clearance increases in response to endurance training in rats (Donovan and Brooks, 1983). An assumption of the model is that muscular energy metabolism controls itself *via* autoregulatory mechanisms. This means that exercise-modulated metabolites such as ADP activate or inhibit key regulatory enzymes such as phosphofructokinase and the pyruvate dehydrogenase complex (Baker et al., 2010). In Mader’s model, ADP and AMP activate metabolic enzymes whereas hydrogen ions and ATP inhibit them (Mader, 2003). Mader’s challenge was to come up with a set of equations that could realistically model the rates of these reactions and the concentration of metabolites depending on an individual’s variables such as muscle mass, maximal glycolytic rate (termed by Mader the maximal rate of lactate formation, abbreviated as ν_La.max_), and oxidative capacity. A comparatively simple task was to simulate the relationship between ATP, ADP, AMP, and phosphocreatine *via* reactions such as the Lohmann reaction as McGilvery and Murray had already modeled this in the mid-1970s (McGilvery and Murray, 1974).

**FIGURE 2:**
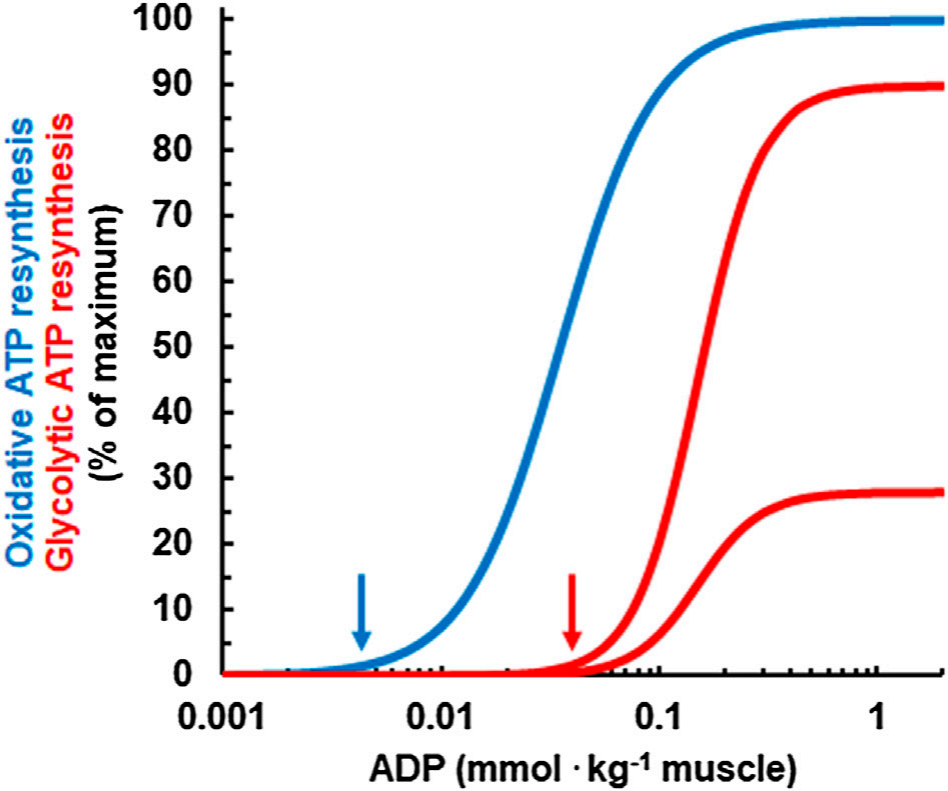
Relationship between the concentration of ADP and the simulated, relative rates of oxidative (blue) and glycolytic ATP resynthesis (red) for a pH of 7 (upper red curve) and for a pH of 6.4 (lower red curve). Note that ADP needs to rise to higher concentrations for glycolytic ATP resynthesis to kick in than for oxidative ATP resynthesis. This is an important feature for the regulation of human energy metabolism and explains why oxidative ATP resynthesis is dominant at rest and during low intensity exercise. Moreover, as an acid pH inhibits phosphofructokinase, the rate of glycolytic ATP resynthesis at a given ADP concentration is lower if the pH is in reduced. In the model, the maximal rate of glycolytic ATP resynthesis is reached at a pH of 7.4 which is not shown in the figure.

Next, Mader had to calculate ATP resynthesis *via* glycolysis based on studies that identified regulators of glycolysis. To model this, Mader assumed ADP and AMP as activators and a low pH as an inhibitor of glycolytic ATP resynthesis (Mader, 1984; Mader, 2003), red curves in Figure 2. This glycolytic ATP resynthesis equation models two important features of exercise metabolism:

1) It models that at low intensities, the rate of pyruvate synthesis by glycolysis is below the rate of pyruvate oxidation, that is, theoretically possible at such intensities. At exercise intensities above the maxLass, glycolytic pyruvate synthesis exceeds the amount of pyruvate that can be oxidized by mitochondria. Therefore, pyruvate and lactate, which are linked *via* the lactate dehydrogenase reaction, rise continuously.
2) It models inhibition of glycolytic ATP resynthesis by a low, acidic pH. Mechanistically, an acid pH inhibits phosphofructokinase which is the rate-limiting enzyme of glycolysis (Trivedi and Danforth, 1966; Dobson et al., 1986).

Finally, Mader used the concentration of ADP to calculate the rate of ATP resynthesis from oxidative phosphorylation. In this model, the activation of oxidative metabolism is described by a Hill equation of 2nd order (blue curve in Figure 2), while the activation of glycolysis is described by a 3rd order equation. Such a sigmoid equation was first used by Archibald V. Hill to describe oxygen binding to hemoglobin (Hill, 1910). Mader deemed this necessary to model “realistic” rates of oxidative ATP resynthesis and V̇O_2_. Later, other studies experimentally confirmed a sigmoid equation for the regulation of oxidative ATP resynthesis (Jeneson et al., 1996; Cieslar and Dobson, 2000).

**FIGURE 3:**
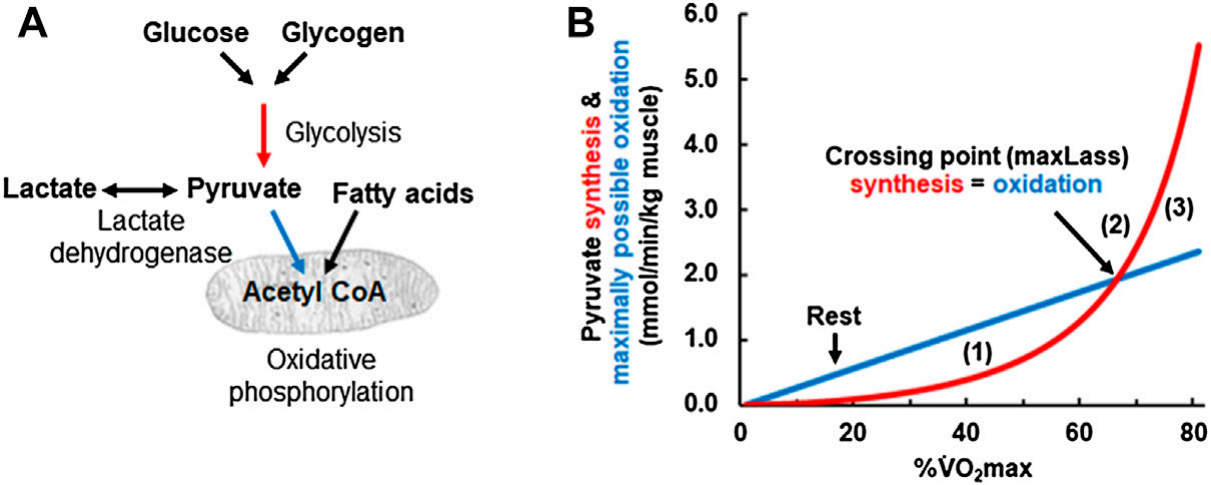
**(A)** Schematic drawing of the pathways simulated in Panel 3 **(B)**. Red arrow refers to glycolytic ATP resynthesis or pyruvate synthesis, the glycolytic flux rate νLa. The blue arrows refer to the oxidative ATP resynthesis or the maximally possible rate of pyruvate oxidation, the oxidative flux (V̇O_2_). **(B)** Calculated glycolytic pyruvate (and lactate) synthesis [red arrow in **(A)** and red line in **(B)**] and maximally possible rate of pyruvate (and lactate) oxidation at different exercise intensities expressed as a percentage of the $\dot{V}$ O_2max_. (1) is the zone where glycolytic pyruvate synthesis is below the maximally possible rate of pyruvate oxidation by oxidative phosphorylation. In this area, the pyruvate deficit allows either fat oxidation to fuel the substrate demand of oxidative metabolism or the uptake of pyruvate *via* previously accumulated lactate. Point (2) is the crossing point where glycolytic pyruvate and lactate synthesis match the maximally possible rate of pyruvate oxidation at a given workload. This point indicates the maxLass. In contrast, (3) is the zone glycolytic flux and the resulting pyruvate synthesis exceeds the maximally possible rate of pyruvate oxidation. As a consequence, pyruvate and lactate accumulate.

In the “engine room” of Mader’s human exercise metabolism model lies the “crossing point” where pyruvate synthesis equals pyruvate oxidation. Figure 3A illustrates the relevant metabolic pathways and Figure 3B the crossing point curves. At low exercise intensities, the concentration of ADP is still comparatively low. Based on the equation for glycolytic ATP resynthesis, the rate of glycolytic pyruvate synthesis (red line in Figure 3B) is lower than the amount of pyruvate that can be oxidized by mitochondria (blue line in Figures 3B–[1]). At such low intensities, glycolysis synthesizes less pyruvate and acetyl-CoA than the mitochondria need for oxidative phosphorylation (i.e., the red pyruvate synthesis line is below the blue pyruvate oxidation line) and so the model assumes that the “missing” acetyl-CoA is synthesized mainly from acetyl CoA generated by the β-oxidation of fatty acids. We discuss this assumption below.

At higher exercise intensities, the rate of glycolysis rises to a point where the rate of pyruvate formation by glycolysis equals the rate of pyruvate oxidation (i.e., the red pyruvate synthesis line meets the blue pyruvate oxidation line in Figures 3B–[2]). At the crossing point, pyruvate synthesis reaches the maximally possible rate of pyruvate oxidation by mitochondria. As pyruvate is in equilibrium with lactate *via* the lactate dehydrogenase reaction, the turning point for pyruvate will be the turning point for lactate and the maxLass (Heck et al., 1985). If the power output rises further, then pyruvate synthesis exceeds the maximally possible rate of pyruvate oxidation and so both pyruvate and lactate will rise with time (Figures 3B–[3]).

The “crossing point” figure also gives a simple explanation for the regulation of fat oxidation, that is, consistent with current thought. To the left of the “crossing point” in Figure 3B, glycolytic pyruvate synthesis is insufficient to provide all of the acetyl-CoA needed for oxidative ATP resynthesis (i.e., the red line is below the blue line). Already in 1986, Mader and Heck proposed that “*the lack of pyruvate* [due to insufficient pyruvate synthesis by glycolysis] *is covered by fatty acid oxidation*” (Mader and Heck, 1986). This is consistent with the observation that fat oxidation is highest at low and medium intensity (i.e., intensity to the left of the crossing point or maxLass) but approaches zero at intensities at and above the maxLass (van Loon et al., 2001). This interpretation also agrees with the recent conclusion by the Kiens group that “*the rate of glycolysis seems thus to be central to mitochondrial acetyl-CoA availability and the regulation of fatty acid oxidation*” (Lundsgaard et al., 2018). In summary, Mader designed already in the 1980s a mathematical model of human metabolism that realistically simulates human metabolic responses to exercise and explains the metabolic basis for the maxLass and the regulation of fat metabolism.

In this review, we will not attempt to provide evidence for all the assumptions underlying Mader’s original and extended model as this has been done elsewhere (Mader, 1984; Mader and Heck, 1986; Mader, 2003). Instead, we will use computer simulations made with a current version of Mader’s human exercise model to both demonstrate that it sufficiently models known human exercise phenomena and to highlight insights that can be gained by its use. First, we will show that Mader’s model simulates the V̇O_2_ slow component and thereby provides a simple, mechanistic explanation for it. Second, we will use the simulations to suggest that a high maximal glycolytic rate ν_La.max_ results in a lower power at the maxLass and reduces endurance performance compared to an athlete with the same $\dot{V}$ O_2max_ but lower ν_La.max_. Third, we will show that a high ν_La.max_ is required to cycle 1,000 m in ≈1 min which is an elite performance.

## Simulation of the Slow Component of the $\dot{V}$ O_2_

**FIGURE 4:**
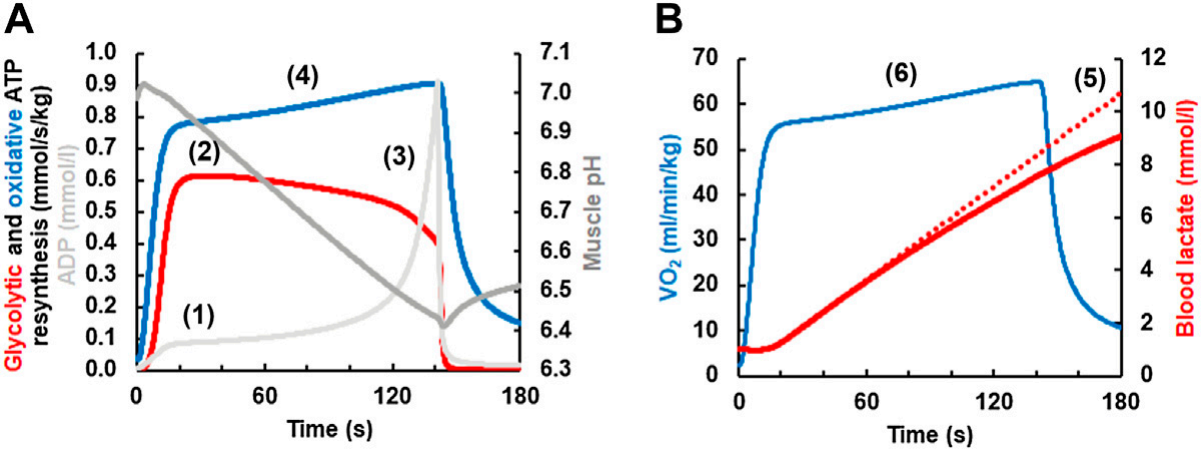
**(A)** Simulation of the V̇O_2_ slow component simulated with a current version of the Mader human exercise metabolism model. At the onset of exercise, the concentrations of ADP and AMP (not shown) increase **(1)**. This stimulates phosphofructokinase and thereby increases the rate of glycolytic ATP resynthesis and lactate formation, reaching a maximum in-between 20–40 s **(2)**. As the concentration of muscle lactate increases **(B)**, muscle pH becomes increasingly acid from ≈20 s onwards **(3)**. As an acid pH inhibits phosphofructokinase (Panel 4A) and thereby glycolytic ATP resynthesis, the rate of glycolytic ATP resynthesis declines after its maximum in-between ≈ 20–40 s even though the concentrations of ADP and AMP (not shown) rise greatly just before fatigue. Because glycolytic ATP resynthesis declines, oxidative ATP resynthesis must rise to meet the constant ATP hydrolysis during constant load exercise **(4)** (the contribution of ATP resynthesis from phosphocreatine is incorporated in the model but not shown). If the efficiency would worsen, then the slow component would increase further. Panel 4B On a whole body level, the reduction in the rate of glycolytic ATP resynthesis and lactate formation is seen **(5)** as a less than linear increase of the lactate concentration (the dotted line plots an increase of lactate at the rate that is reached in-between 20–40 s). The slow component of oxidative ATP resynthesis in **(A)** is seen as a corresponding slow component of the V̇O_2_ in **(B) (6)**.

In 1986, Brian Whipp and Karlman Wasserman described a “*second, slower component of $\dot{V}$ O*_*2*_” at the onset of constant load exercise especially at intensities exceeding the maxLass (Whipp and Wasserman, 1986). The slow component of the V̇O_2_ has stimulated much research to try to identify the mechanisms that cause it. Jones et al. have reviewed this research and conclude that the $\dot{V}$ O_2_ slow component “*represents a progressive loss of skeletal muscle contractile efficiency and is associated with the fatigue process*” (Jones et al., 2011). Mader’s model already in its version of 1984 simulated the $\dot{V}$ O_2_ slow component based on the following set of mechanisms (see Figure 4):

1) During exercise above the maxLass the lactate concentration increases continuously in skeletal muscle and blood.
2) Because of the rise of lactate, the pH will decrease.
3) A low pH inhibits phosphofructokinase (Trivedi and Danforth, 1966; Dobson et al., 1986) and thereby reduces the rate of glycolytic ATP resynthesis and lactate formation after its initial peak.
4) If the ATP cost of exercise remains constant, then the pH-dependent reduction of glycolytic ATP resynthesis must be compensated by a rise in the rate of oxidative ATP resynthesis which manifests itself as the $\dot{V}$ O_2_ slow component.
5) If efficiency would worsen (Jones et al., 2011), which is not modeled by Mader’s model, then the $\dot{V}$ O_2_ slow component would be even larger.

Recent studies concluded that the rate of glycolytic ATP resynthesis decreases with time during exercise at an intensity higher than the maxLass, as modeled in Figure 4. For example, O’Connell and colleagues suggested “*that the rate of ATP replenishment by anaerobic metabolism might actually continue to decrease after the time when a steady state for V̇O*_*2*_ *should be achieved*” (O’Connell et al., 2017). Similarly, Colosio et al. also suggested that “*the emergence of a V̇O*_*2*_ *slow component is ascribable to a* ‘*metabolic shift*’ *between aerobic and anaerobic metabolisms*” (Colosio et al., 2020), which is what Mader`s model essentially predicted in the mid-1980s.

## The Maximal Rate of Glycolysis or Lactate Formation (ν_La.max_) and Its Influence on the maxLass

Intuitively, one might assume that the ultimate endurance athlete would combine a high glycolytic and oxidative ATP resynthesis. However, glycolysis is a self-limiting system as it causes lactate to accumulate which in turn will cause the pH to fall which will inhibit phosphofructokinase and glycolytic ATP resynthesis (Figure 3) (Trivedi and Danforth, 1966; Dobson et al., 1986). Moreover, Mader`s model predicts that athletes with a higher ν_La.max_ generally have higher lactate concentrations at the same workload, reach their maxLass at a lower workload and, due to higher carbohydrate usage, perform less well at long distance endurance events even if their $\dot{V}$ O_2max_ is the same as in an athlete with a lower ν_La.max_. The inverse relationship between the ν_La.max_ and the maxLass has been previously reported for runners and in cyclists (Joyner and Coyle, 2008).

**FIGURE 5:**
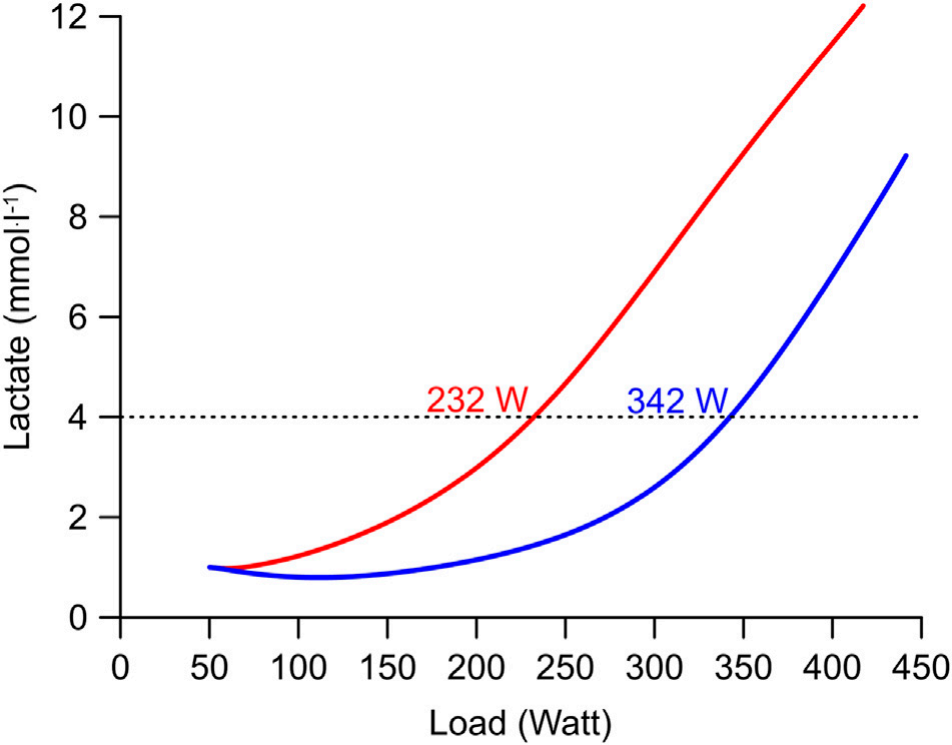
Simulation of a graded exercise test of two subjects with extreme differences of their ν_La.max_ but equal $\dot{V}$ O_2max_ (65 ml min^−1^ kg^−1^). The lactate curves are simulated using the revised version of Mader’s human exercise metabolism model (Mader, 1984; Mader, 2003). The first subject (blue curve) has a low maximal glycolytic rate (ν_La.max_) of 0.3 mmol^.^L^−1^ s^−1^. The second subject (red curve) has a ν_La.max_ of 1.0 mmol^.^L^−1^ s^−1^.

Using Mader’s equations, we have modeled a graded exercise test of two athletes, both with a $\dot{V}$ O_2max_ of 65 ml min^−1^ kg^−1^ (Figure 5). The first athlete (blue line) has a low maximum glycolytic rate (ν_La.max_) and can maximally synthesize 0.3 mmol of lactate per liter and second. In contrast, the other athlete has a 3 times higher ν_La.max_ and can maximally synthesize 1.0 mmol of lactate per liter and second. Such high values of the ν_La.max_ (i.e. >1.0 mmol^.^L^−1.^ s^−1^) are typical for sprinters. The examples used in our simulations were chosen to demonstrate the effect of the individual ν_La.max_ on the 4 mmol^.^L^−1^ lactate threshold at an identical $\dot{V}$ O_2max_. Unsurprisingly, the athlete with the higher ν_La.max_ (red line in Figure 5) synthesizes more lactate also at a lower power output because the same ADP and AMP concentrations will stimulate more phosphofructokinase molecules. Therefore, the lactate concentration is higher at every workload. As a consequence, the model predicts that the athlete with the high ν_La.max_ has a 110 W lower workload at a 4 mmol^.^L^−1^ lactate threshold (Figure 5) than the athlete with the low ν_La.max_. Specifically, in constant pace long-term endurance events where sprinting ability matters little (e.g., marathon, Olympic distance, and Ironman triathlon), athletes should aim for a minimal glycolytic ATP resynthesis capacity.

The idea that a high ν_La.max_ can hinder endurance performance is consistent with the observation that endurance athletes have high percentages of type 1 fibers (Costill et al., 1976; Gehlert et al., 2012), which usually contain low concentrations of anaerobic enzymes (Murgia et al., 2017). Because of these insights, some sports scientists and practitioners especially in German-speaking countries now consider the ν_La.max_ as an important variable when analysing the metabolism of an endurance athlete (Weber, 2003; Nitzsche et al., 2018).

Finally, a rightward shift of the lactate curve after, for example, several weeks of endurance training (see e.g., Figure 1) can potentially be explained either by a drop of the ν_La.max_ or by an increase of lactate oxidation, or both. Here, George Brooks and coworkers have demonstrated early on that the rate of lactate oxidation increases after endurance training (Donovan and Brooks, 1983) whereas the ν_La.max_ does not decrease (Hommel et al., 2019). Having said that, chronic 10 Hz electrical stimulation of a rabbit fast-twitch tibialis anterior muscle reduces the expression of glycolytic enzymes, such as phosphofructokinase (Henriksson et al., 1986), which will reduce the ν_La.max_. Therefore, the rightward shift of the lactate curve after endurance training is currently mainly explained by an increase of lactate oxidation but more research is needed to find out whether changes of the ν_La.max_ can contribute to the rightward shift of the lactate curve.

## A High ν_La.max_ Is Required for High Power Output During Short-Term Exercise

**FIGURE 6:**
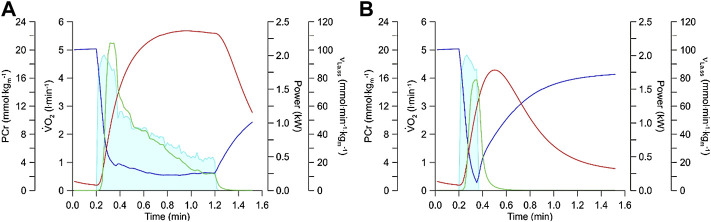
**(A)** Simulation of energy metabolism during a 1,000 m time trial of a track cyclist. This calculation is based on the recorded power output from an Olympic track cyclist using the equations from the Mader model, the computer simulation calculates the behavior of glycolytic flux (green line; expressed per kg muscle), PCr levels (blue line), and oxygen uptake (red line) in dependency of the power output (light blue shade). Physiological variables used in the simulation were $\dot{V}$ O_2max_: 65 ml min^−1^ kg^−1^; ν_La.max_: 0.9 mmol^.^L^−1^ s^−1^; bodyweight: 95 kg; and active muscle mass: 40%. **(B)** Simulation of energy metabolism for the power output during the 1,000 m time trial as conducted by the athlete in Panel 6A but possessing a lower maximal glycolytic rate. The input parameters were $\dot{V}$ O_2max_: 65 ml min^−1^ kg^−1^; ν_La.max_: 0.4 mmol^.^L^−1^ s^−1^; bodyweight: 95 kg; and active muscle mass: 40%.

In many sports, power outputs that exceed 1000 W for several seconds to more than a minute are required (Sahlin, 2014). Such disciplines include games such as football, handball, basketball or rugby, martial arts, 400 m sprints and for example, 1,000 m track cycling. To determine how a high ν_La.max_ affects performance in such events, we have simulated the metabolic response to the measured power output of an elite track cyclist who cycled 1,000 m in a time of 1 min and 1 s with a mean power output of 950 watts and a starting power output of nearly 2000 watts. Figure 6A shows a simulation for a relative $\dot{V}$ O_2max_ of 65 ml min^−1^ kg^−1^ and a ν_La.max_ of 0.9 mmol^.^L^−1^ s^−1^. With these input data, the model predicts that the athlete can achieve this “real world” performance. In Figure 6B, the ν_La.max_ is reduced to 0.4 mmol^.^L^−1^ s^−1^ and the simulation predicts that the athlete with the lower ν_La.max_ is unable to sustain the “real world” power output for the full 1,000 m.

The simulation demonstrates the importance of a high ν_La.max_ (which is essentially a high activity of glycolytic enzymes) for events where the power output reaches very high values for a few seconds to just more than a minute in elite athletes.

## The Challenge of Measuring the ν_La.max_

One insight from Mader’s model is that the ν_La.max_ is an important, additional variable that influences endurance performance. Mader proposed to estimate the maximum rate of glycolysis by lactate measurements before and after an all-out sports-specific sprint test using the following equation (Mader, 1984):

$${\text{ν}}_{\mathrm{La}.\text{max}}=\frac{\max\left[\text{LA}\right]\text{ post}-\left[\text{LA}\right]\text{rest}}{t\;ex-t\;alac},$$

where ${\text{ν}}_{\mathrm{La}.\text{max}}$ = maximal glycolytic rate or maximal lactate synthesis rate (mmol s^−1^ l^−1^), max [La] _post_ = maximal lactate concentration after exercise (mmol l^−1^), [La]_rest_ = lactate concentration at rest (mmol l^−1^), t _ex_ = exercise duration (s), and t _alac_ = time (s) equivalent to account for ATP resynthesis from phosphocreatine.

In practice, measuring the ν_La.max_ is challenging. The test setup must allow on the one hand an effort that maximally activates glycolysis during, for example, a 10 s effort while on the other hand limiting the activation of oxidative metabolism as much as possible to reduce intramuscular lactate oxidation (Heck et al., 2003).

Further considerations concerning the ν_La.max_ assume that individuals with a higher amount of muscle mass and more fast, glycolytic type 2A and 2X muscle fibers produce generally higher lactate levels during aerobic and anaerobic exercise than subjects with fewer type 2A and 2X fibers (Ivy et al., 1980). Importantly, those athletes produce more lactate at every exercise intensity and reach higher maximal lactate concentrations in a graded exercise test (Mader and Heck, 1986; Mader, 2003). As a result, the exercise intensity at maxLass is lower and absolute carbohydrate usage higher, which is detrimental during long distance endurance events (Jones, 2006). In contrast, athletes participating, for example, in glycolytic and intermittent sports, must rely on a high glycolytic in combination with a high oxidative capacity.

## Utility of the Mader Model

To us, Mader’s model of human energy metabolism was far ahead of its time when it was published in 1984 and is has arguably stood the test of time. But what are its benefits? First, Mader’s model gives a plausible, mechanistic explanation of how human exercise metabolism works. This is, for example, evident from the simulation of the $\dot{V}$ O_2max_ slow component, that is, in line with recent reports (Figure 4). This helps users to understand the causes of metabolic phenomena during exercise. Second, it allows users to identify through simulations the $\dot{V}$ ;O_2max_, ν_La.max_, or muscle mass necessary to achieve a desired performance. This is illustrated in Figure 6 and helps to set targets for a training plan. Third, it allows to simulate % or gram fat and % or gram carbohydrate oxidation for a given exercise which is useful for, for example, planning nutrient intake during an endurance event. Fourth, it has highlighted that the ν_La.max_ influences the lactate curve (Figure 5). Based on the insight that a high ν_La.max_ impairs endurance performance, long-distance endurance athletes should avoid training that increases the amount and activity of glycolytic enzymes in their musculature to avoid a resultant leftward shift of the workload lactate curve. The problem, however, is that the ν_La.max_ is hard to measure in practice.

## Summary and Conclusion

In summary, the Cologne group has introduced and simplified lactate testing, defined the 4 mmol^.^L^−1^ lactate threshold, maxLass and ν_La.max_, and has generated the first, comprehensive simulation model of human exercise metabolism. Some of these conceptual advances are underappreciated outside German-speaking countries especially because some seminal publications are not easily accessible. We therefore hope that this article will help to raise the awareness of the Cologne group and their contributions.

## Dedication

On the 13.5.2021, Prof Dr Wildor Hollmann passed away. He founded the Institute of Sports Medicine in Cologne in 1958, led the Cologne group and motivated and taught generations of students who loved his lectures. Prof. Dr. Claude Bouchard considers Wildor Hollmann as his first mentor (Bouchard, 2022). As students of Wildor Hollmann, we dedicate this article to his memory.
